# Omega-3 Fatty Acids Protect Renal Functions by Increasing Docosahexaenoic Acid-Derived Metabolite Levels in SHR.Cg-*Lepr^cp^*/NDmcr Rats, a Metabolic Syndrome Model

**DOI:** 10.3390/molecules19033247

**Published:** 2014-03-17

**Authors:** Masanori Katakura, Michio Hashimoto, Takayuki Inoue, Abdullah Al Mamun, Yoko Tanabe, Ryo Iwamoto, Makoto Arita, Satoru Tsuchikura, Osamu Shido

**Affiliations:** 1 Department of Environmental Physiology, Shimane University Faculty of Medicine, Izumo, Shimane 693-8501, Japan; E-Mails: katakura@med.shimane-u.ac.jp (M.K.); tinoue@med.shimane-u.ac.jp (T.I.); mamun104@gmail.com (A.M.); tanabey@med.shimane-u.ac.jp (Y.T.); o-shido@med.shimane-u.ac.jp (O.S.); 2 Department of Developmental Biology, Shimane University Faculty of Medicine, Izumo, Shimane 693-8501, Japan; 3 Department of Health Chemistry, Graduate School of Pharmaceutical Sciences, The University of Tokyo, 7-3-1 Hongo, Bunkyo-ku, Tokyo, 113-0033, Japan; E-Mails: riwamoto@mol.f.u-tokyo.ac.jp (R.I.); marita@mol.f.u-tokyo.ac.jp (M.A.); 4 Disease Model Cooperative Research Association, Hamamatsu, Shizuoka, 433-8114, Japan; E-Mail: shrtsuchikura@h5.dion.ne.jp

**Keywords:** protectin, resolvin, lipidomics, diabetic nephropathy

## Abstract

The omega-3 polyunsaturated fatty acids (ω-3 PUFAs) docosahexaenoic acid (DHA) and/or eicosapentaenoic acid (EPA) protect against diabetic nephropathy by inhibiting inflammation. The aim of this study was to assess the effects of highly purified DHA and EPA or EPA only administration on renal function and renal eicosanoid and docosanoid levels in an animal model of metabolic syndrome, SHR.Cg-*Lepr^cp^*/NDmcr (SHRcp) rats. Male SHRcp rats were divided into 3 groups. Control (5% arabic gum), TAK-085 (300 mg/kg/day, containing 467 mg/g EPA and 365 mg/g DHA), or EPA (300 mg/kg/day) was orally administered for 20 weeks. The urinary albumin to creatinine ratio in the TAK-085-administered group was significantly lower than that in other groups. The glomerular sclerosis score in the TAK-085-administered group was significantly lower than that in the other groups. Although DHA levels were increased in total kidney fatty acids, the levels of nonesterified DHA were not significantly different among the 3 groups, whereas the levels of protectin D1, resolvin D1, and resolvin D2 were significantly increased in the TAK-085-administered group. The results show that the use of combination therapy with DHA and EPA in SHRcp rats improved or prevented renal failure associate with metabolic syndrome with decreasing triglyceride levels and increasing ω-3 PUFA lipid mediators.

## 1. Introduction

The omega-3 polyunsaturated fatty acids (ω-3 PUFAs) docosahexaenoic acid (DHA) and/or eicosapentaenoic acid (EPA) protect against ischemic acute renal failure [1], IgA- [2,3,4] or cyclosporine A-induced nephrotoxicity [5,6], and streptozocin-induced type 1 diabetic [7] and, type 2 diabetic nephropathy [8,9,10,11] in rodents. These renal failures are closely related with inflammation, which promotes the loss of renal function. Inflammatory cytokines and reactive oxygen species (ROS) activate arachidonic acid (ARA) release from kidney cell membranes. Huang *et al.* reported that interleukin-1 rapidly stimulates the release of phospholipase A_2_ (PLA_2_) activity-dependent ARA and activates mesangial cells via the Jun N-terminal/stress-activated protein kinase signaling pathway [12]. ROS activate renal mitochondrial PLA_2_ activity and cyclooxygenase-2 (COX-2) expression in the kidney [13,14]. The effects of tumor necrosis factor-α on ion transport are related to the induction of COX-2-dependent prostaglandin (PG) E_2_ synthesis [15].

DHA and EPA have various anti-inflammatory effects. These fatty acids are oxidized by COXs, lipoxygenases, or cytochrome P450 monooxygenases to produce docosanoids and EPA-derived eicosanoids, which have anti-inflammatory effects [16]. Dietary ω-3 fatty acids are considered to prevent inflammation through a variety of activities linked to the inhibition of ARA-derived eicosanoid-mediated effects, anti-inflammatory properties, and competitive inhibition of cytokines and ARA-derived eicosanoid synthetic enzymes. ω-3 PUFA-derived resolvins (Rvs) and protectins (PDs) inhibit neutrophil infiltration into injured kidneys, block toll-like receptor-mediated inflammatory activation of macrophages, and mitigate renal function [17]. TAK-085, a concentrated formulation of ω-3 PUFAs, attenuates albuminuria and renal dysfunction with a decrease in sterol regulatory element-binding protein (SREBP)-1 expression and triglyceride (TG) levels in the kidney of type 2 diabetic db/db mice [8]. However, the effects of ω-3 PUFAs on renal eicosanoid and docosanoid levels in metabolic syndrome model animals have not been reported.

The aim of this study was to assess the effects of highly purified DHA and EPA or EPA only administration on renal function in an animal model of metabolic syndrome, SHR.Cg-*Lepr^cp^*/NDmcr (SHRcp) rats. SHRcp rats were chosen because they exhibit several metabolic disorders, such as hypertension, hyperglycemia, hyperinsulinemia, and hyperlipidemia [18]. Islet area expansion, fatty liver, and glomerulosis can be histologically observed in these rats. Therefore, they are a suitable animal model for renal dysfunction with metabolic syndrome. In this study, we measured renal eicosanoid and docosanoid levels using liquid chromatography–electrospray ionization/tandem mass spectrometry (LC–ESI–MS/MS) because long-term administration of ω-3 PUFAs may affect the levels of eicosanoids and docosanoids. Changes in the levels of eicosanoids and docosanoids modulate renal function and inflammatory status, as described above.

## 2. Results and Discussion

### 2.1. Effects of ω-3 PUFAs on Renal Function in the SHRcp Rats

#### 2.1.1. Body Weight and Blood Pressure

ω-3 PUFA administration did not affect body weight or blood pressure of SHRcp rats throughout the experimental period (Table 1). Serum creatinine, blood urea nitrogen, TG, and total cholesterol (TC) levels did not significantly change among the groups.

**Table 1:** Effects of TAK-085 and EPA administration on body weight, blood pressure, and plasma biological parameters.

Parameter	Control	TAK-085	EPA
Body weight (g)	591.5 ± 6.1	614.0 ± 6.9	595.6 ± 6.6
SBP (mmHg)	199.5 ± 4.5	192.9 ± 4.2	200.5 ± 3.6
MBP (mmHg)	165.5 ± 3.7	160.3 ± 3.1	163.3 ± 2.4
DBP (mmHg)	153.5 ± 3.8	145.7 ± 4.2	148.3 ± 3.4
Creatinine (mg/dL)	0.27 ± 0.01	0.30 ± 0.01	0.30 ± 0.01
BUN (mg/dL)	30.3 ± 0.9	31.5 ± 0.8	34.9 ± 1.3
Triglyceride (mg/dL)	266.9 ± 32.3	253.8 ± 13.1	341.3 ± 33.7
Total cholesterol (mg/dL)	368.3 ± 26.3	379.5 ± 10.4	422.6 ± 19.8
HDL-cholesterol (mg/dL)	51.3 ± 1.1	52.1 ± 1.2	55.5 ± 0.8

*Notes*: SBP; Systolic blood pressure, MBP; mean arterial blood pressure, DBP; diastolic blood pressure, BUN; blood urea nitrogen, HDL; high density lipoprotein. Values are means ± SE for 10 or 11 rats. Statistical analysis was performed by one-way ANOVA followed by Dunnett’s test. *, *P* < 0.05 *vs.* control group.

#### 2.1.2. Renal Function Parameters

**Figure 1:**
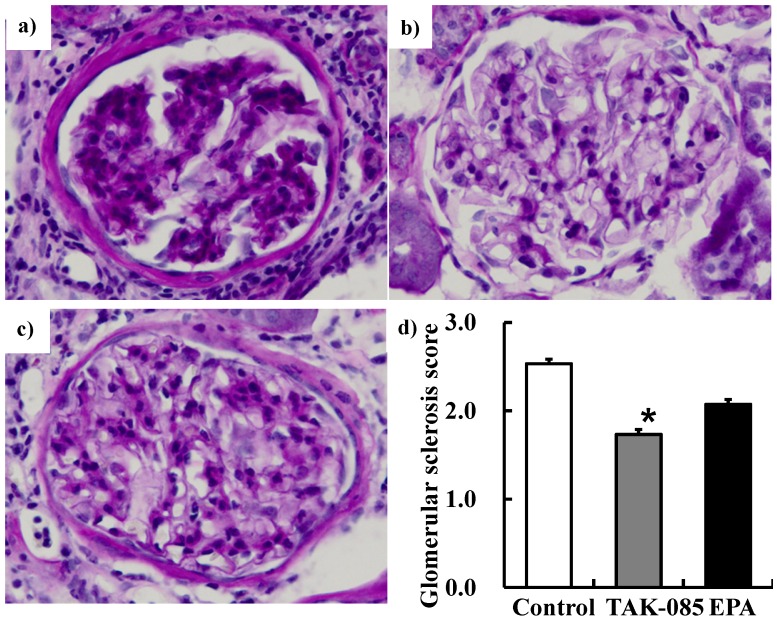
Photomicrographs of coronal sections of the glomeruli from (**a**) the control group, (**b**) the TAK-085-administered group, (**c**) and the EPA-administered group. (**d**) Glomerulosclerosis was semi-quantitatively evaluated. Values are presented as mean ± SE (n = 10–11). Statistical analysis was performed by one-way ANOVA followed by Dunnett’s test. * *P* < 0.05 *vs.* control group.

ROS and lipid peroxide levels in the kidney were not significantly different among the 3 groups (Data were not shown). Urinary albumin tended to be lower, but not significantly, in the TAK-085-administered group than that in the other groups, leading to an albumin to creatinine ratio of 36.3% in the TAK-085-administered group, which was significantly lower than that in the other groups. Creatinine clearance increased in the TAK-085-administered group (Table 2). TAK-085 administration inhibited histological damage to the kidneys. The glomerular sclerosis score was significantly lower in the TAK-085-administered group than that in the other groups (Figure 1).

### 2.2. Effects of ω-3 PUFAs on Lipid Levels in SHRcp Rats

#### 2.2.1. Plasma, Kidney, and Liver Fatty Acid Profiles

Plasma fatty acid profiles after 20 weeks of administration are shown in Table 3. Mole percentages of palmitic acid (PLA) decreased significantly in the EPA-administered group. Mole percentages of oleic acid (OA) and ARA decreased significantly and mole percentages of linoleic acid (LA), EPA, docosapentaenoic acid (DPAn-3), and DHA increased significantly in the TAK-085- and EPA-administered groups. The DHA/ARA and EPA/ARA ratios increased significantly, whereas the n-6/n-3 ratio decreased in the TAK-085- and EPA-administered groups.

**Table 2:** Effects of TAK-085 and EPA on renal functions.

	Control	TAK-085	EPA
Water intake (mL/kg bw. day)	64.9 ± 4.3	56.1 ± 3.3	61.2 ± 1.9
Urine flow (mL/kg bw. day)	36.8 ± 1.5	32.1 ± 2.4	37.5 ± 1.7
Urinary albumin (mg/kg bw. day)	163.6 ± 11.7	134.1 ± 12.1	172.5 ± 12.6
Urinary creatinine (mg/kg bw. day)	6.9 ± 0.7	8.9 ± 0.6	7.3 ± 0.6
AC ratio	25.7 ± 2.6	16.3 ± 2.4 *	28.5 ± 6.0
Creatinine clearance (mL/min)	0.88 ± 0.08	1.27 ± 0.07 *	0.96 ± 0.11

*Notes*: AC ratio; urinary albumin to urinary creatinine ratio. Values are mean ± SE (n = 10–11). Statistical analysis was performed by one-way ANOVA followed by Dunnett's test. *, *P* < 0.05 *vs.* control group.

Fatty acid profiles in the liver after 20 weeks of administration are shown in Supplemental Table 1. Mole percentages of PLA, OA, and ARA decreased significantly in the TAK-085- and EPA-administered groups. Mole percentages of LA, α-linolenic acid, EPA, DPAn-3, and DHA increased significantly in the TAK-085- and EPA-administered groups. The DHA/ARA and EPA/ARA ratios increased significantly, whereas the n-6/n-3 ratio decreased in the TAK-085- and EPA-administered groups.

**Table 3:** Effects of TAK-085 and EPA on fatty acid profiles in plasma.

(mol%)	Control	TAK-085	EPA
PLA (16:0)	26.03 ± 0.16	25.73 ± 0.21	25.43 ± 0.12 *
STA (18:0)	6.63 ± 0.10	6.23 ± 0.06	6.32 ± 0.09
OA (18:1n-9)	25.20 ± 0.40	23.33 ± 0.17 *	23.47 ± 0.27 *
LA (18:2n-6)	18.45 ± 0.17	20.99 ± 0.24 *	19.77 ± 0.13 *
ALA (18:3n-3)	0.50 ± 0.02	0.58 ± 0.02 *	0.59 ± 0.02 *
ARA (20:4n-6)	19.48 ± 0.48	11.14 ± 0.30 *	11.83 ± 0.31 *
EPA (20:5n-3)	0.69 ± 0.04	4.11 ± 0.18 *	6.89 ± 0.15 *
DPA (22:5n-3)	1.00 ± 0.02	1.51 ± 0.02 *	3.24 ± 0.05 *
DHA (22:6n-3)	1.36 ± 0.08	5.73 ± 0.11 *	1.81 ± 0.03 *
n-6/n-3	10.80 ± 0.39	2.71 ± 0.07 *	2.53 ± 0.05 *
DHA/ARA	0.07 ± 0.01	0.52 ± 0.02 *	0.15 ± 0.01 *
EPA/ARA	0.04 ± 0.00	0.37 ± 0.02 *	0.59 ± 0.03 *
SCD index	3.82 ± 0.11	3.75 ± 0.05	3.72 ± 0.08

*Notes*: PLA, palmitic acid; STA, stearic acid, OA, oleic acid; LA, linolenic acid; ALA, α-Linolenic acid; ARA, arachidonic acid; EPA, eicosapentaenoic acid; DPA, docosapentaenoic acid; DHA, docosahexaenoic acid. SCD index was estimated as ratio of OA to STA. Values are means ± SE for 10–11 rats. *, Statistically significant from control group (*P* < 0.05, Dunnett t-test).

Fatty acid profiles in the kidney after 20 weeks of administration are shown in Table 4. Mole percentages of PLA and ARA decreased significantly in the TAK-085- and EPA-administered groups. Mole percentages of LA, EPA, DPAn-3, and DHA increased significantly in the TAK-085- and EPA-administered groups. The DHA/ARA and EPA/ARA ratios increased significantly, whereas the n-6/n-3 ratio decreased in the TAK-085- and EPA-administered groups.

**Table 4:** Effects of TAK-085 and EPA on fatty acid profiles in the kidney.

(mol%)	Control	TAK-085	EPA
PLA (16:0)	28.48 ± 0.14	27.39 ± 0.16 *	27.55 ± 0.17 *
STA (18:0)	14.70 ± 0.31	14.48 ± 0.28	14.10 ± 0.37
OA (18:1n-9)	16.59 ± 0.93	14.38 ± 0.83	16.36 ± 1.05
LA (18:2n-6)	12.17 ± 0.18	15.26 ± 0.15 *	14.38 ± 0.15 *
ALA (18:3n-3)	0.26 ± 0.01	0.24 ± 0.01	0.26 ± 0.01
ARA (20:4n-6)	22.24 ± 0.61	17.37 ± 0.46 *	16.87 ± 0.55 *
EPA (20:5n-3)	0.24 ± 0.02	2.00 ± 0.05 *	2.92 ± 0.11 *
DPA (22:5n-3)	0.74 ± 0.02	1.34 ± 0.04 *	2.70 ± 0.07 *
DHA (22:6n-3)	1.45 ± 0.08	4.15 ± 0.10 *	1.65 ± 0.06
n-6/n-3	12.92 ± 0.36	4.25 ± 0.11 *	4.16 ± 0.07 *
DHA/ARA	0.07 ± 0.01	0.24 ± 0.01 *	0.10 ± 0.01 *
EPA/ARA	0.01 ± 0.01	0.12 ± 0.01 *	0.17 ± 0.02 *
SCD index	1.15 ± 0.09	1.01 ± 0.08	1.19 ± 0.11

Notes: PLA, palmitic acid; STA, stearic acid, OA, oleic acid; LA, linolenic acid; ALA, α-Linolenic acid; ARA, arachidonic acid; EPA, eicosapentaenoic acid; DPA, docosapentaenoic acid; DHA, docosahexaenoic acid. SCD index was estimated as ratio of OA to STA. Values are means ± SE for 10–11 rats. *, Statistically significant from control group (*P* < 0.05, Dunnett t-test).

#### 2.2.2. Kidney and Liver TG and TC Levels

**Figure 2:**
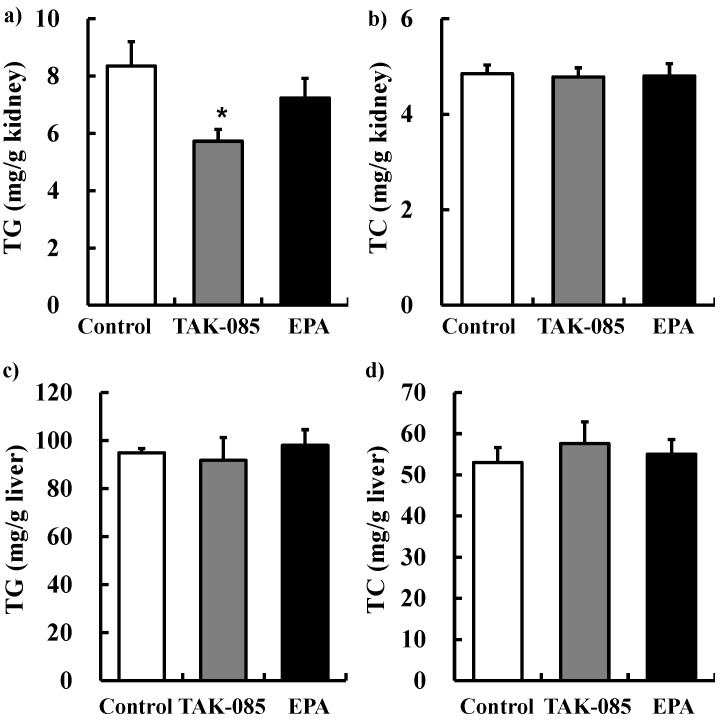
Effects of TAK-085 and EPA on lipid levels in the kidney and liver. TG (**a**) and TC (**b**) levels in the kidney. TG (**c**) and TC (**d**) levels in the liver. Values are presented as mean ± SE (n = 10–11). Statistical analysis was performed by one-way ANOVA followed by Dunnett’s test. * *P* < 0.05 *vs.* control group.

TG and TC levels in the kidney and liver after 20 weeks of administration are shown in Figure 2. TG levels in the kidney decreased significantly in the TAK-085-administered group but not in the EPA-administered group. TC levels in the kidney were not significantly different among the 3 groups.

### 2.3. Levels of Eicosanoids and Docosanoids in the Kidney

**Figure 3:**
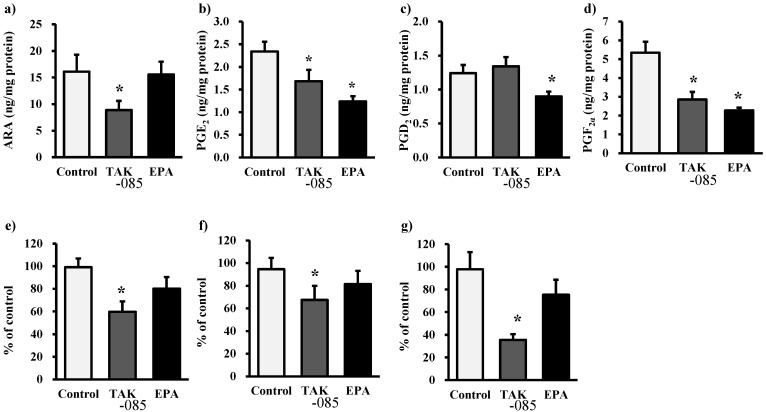
Renal levels of ARA-derived eicosanoids. (**a**) Nonesterified ARA, (**b**) PGE_2_, (**c**) PGD_2_, (**d**) PGF_2α_, (**e**) 5-HETE, (**f**) 12-HETE, and (**g**) 15-HETE. Values are presented as mean ± SE (n = 10–11). Statistical analysis was performed by one-way ANOVA followed by Dunnett’s test. * *P* < 0.05 *vs.* control group.

**Figure 4:**
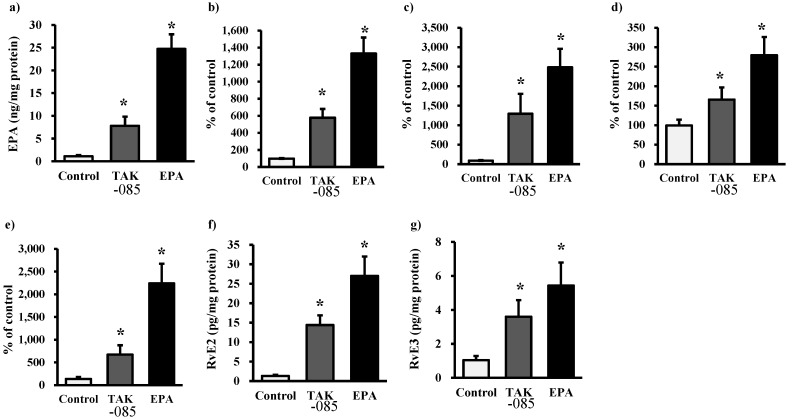
Renal levels of EPA-derived eicosanoids. (**a**) Nonesterified EPA, (**b**) 5-HEPE, (**c**) 12-HEPE, (**d)** 15-HEPE, (**e**) 18-HEPE, (**f**) RvE2, and (**g**) RvE3. Values are presented as mean ± SE (n = 10–11). Statistical analysis was performed by one-way ANOVA followed by Dunnett’s test. * *P* < 0.05 *vs.* control group.

**Figure 5:**
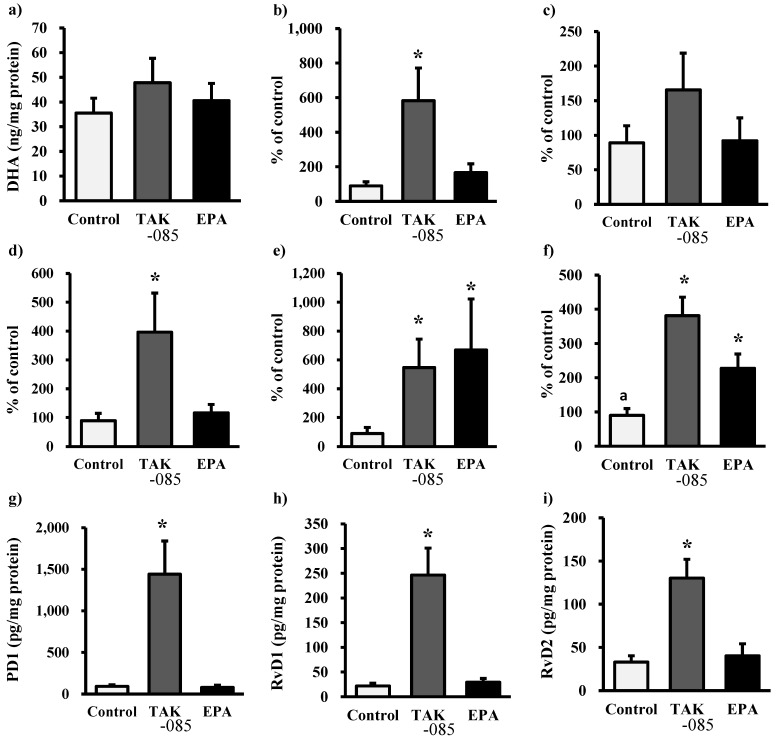
Renal levels of DHA-derived docosanoids. (**a**) Nonesterified DHA, (**b**) 7-HDoHE, (**c**) 13-HDoHE, (**d**) 10-HDoHE, (**e**) 14-HDoHE, (**f**) 17-HDoHE, (**g**) PD1, (h) RvD1, and (**i**) RvD2. Values are presented as mean ± SE (n = 10–11). Statistical analysis was performed by one-way ANOVA followed by Dunnett’s test. * *P* < 0.05 *vs.* control group.

Kidney analyses revealed a decrease in renal formation of PGE_2_ and PGF_2α_ in the TAK-085- and EPA-administered groups. The levels of nonesterified ARA, 5-hydroxyeicosatetraenoic acid (5-HETE), 12-HETE, and 15-HETE decreased significantly in the TAK-085-administered group (Figure 3). The levels of nonesterified EPA and EPA-derived eicosanoid in the kidney increased significantly in the TAK-085- and EPA-administered groups (Figure 4). The levels of nonesterified DHA were not significantly different among the three groups, whereas the levels of 7-hydroxy docosahexaenoic acid (7-HDoHE), 10-HDoHE, PD1, RvD1, and RvD2 increased significantly in the TAK-085-administered group (Figure 5). A significant inverse correlation was found between glomerular sclerosis score and renal PD1 (*r* = −0.720, *P* = 0.029), RvD1 (*r* = −0.771, *P* = 0.0215, and RvD2 (*r* = −0.796, *P* = 0.010).

### 2.4. Discussion

This is the first report to show increased production of RvEs, RvDs, and PD1 in a type 2 diabetic kidney model treated with ω-3 PUFAs, associated in a reduction in functional and morphological renal dysfunction. The anti-inflammatory lipid mediators RvE was produced in the EPA-administered kidney model. Further, administration of TAK-085, combination of EPA with DHA, resulted in the production of RvDs and PD1 in addition to RvEs in the kidney. Moreover, a significant inverse correlation was observed between glomerular sclerosis score and DHA-derived docosanoids (protectin D1, resolvin D1, and resolvin D2) in the kidney. Taken together, our results demonstrate that DHA-derived docosanoids possibly contribute to suppress the progression of renal dysfunction in SHRcp.

TAK-085 but not EPA alone inhibited the progression of renal dysfunction in the SHRcp rats (Table 2 and Figure 1). However, it has been reported that EPA ethyl ester administration improves type 2 diabetic nephropathy in KKA^y^/Ta mice [10]. These conflicting results could be because of the differences in food, EPA dose, or blood pressure. In the present study, a fish oil-deficient diet containing 1% cholesterol and 0.3% cholic acid was used instead of the normal rodent diet. Hypercholesterolemia aggravates nephrotoxicity [19], suggesting that a more severe nephrotoxic model was used in the present study. Although EPA ethyl ester was used in both studies, 300 mg/kg EPA instead of 1000 mg/kg was used with a different route of administration. The systolic blood pressure (SBP) was 200.5 ± 3.6 mmHg in the EPA-administered group (Table 1), whereas it was 116.1 ± 3.9 mmHg in KKA^y^/Ta mice treated with EPA for 20 weeks [10]. Hypertension increases extracellular volume and vasoconstriction, resulting in impaired renal function [20]. Reducing blood pressure with medication remains a primary goal in preventing the incidence of diabetic kidney and slowing its progression. EPA administration did not affect blood pressure in the present study (Table 1) or in a previous study [10]. SBP was higher in the mice in the present study than in KKA^y^/Ta mice, which may have been due to the more severe nephrotoxicity model used in the present study. Taken together, these results suggest that a higher dose of EPA may be necessary to improve renal function in SHRcp rats.

TAK-085 administration increased DHA levels in the plasma, liver, and kidney, whereas EPA administration increased DHA levels in the plasma and liver but not in the kidney. These results suggest that exogenous EPA may be incorporated in the liver and converted to DHA thorough DPAn-3; however, conversion of DPAn-3 to DHA is limited because DPAn-3 levels increased in the kidney of the EPA-administered group (Table S1). It has been reported that Δ6 desaturase activity, which is involved in the conversion of DPAn-3 to DHA, is associated with a higher diabetes risk [21,22]. It is possible that DHA levels in the kidney did not increase in EPA-administered group. It has been reported that high dose of ω-3 PUFAs suppress Δ6 desaturase activity in the liver [23]. Taken together, these results suggest that EPA, a DHA precursor, is insufficient to increase DHA levels in the diabetic kidney because of limited conversion of EPA to DHA in EPA-administered SHRcp rats.

Accumulation of TG in the renal tubules has been observed in cisplatin-induced acute kidney injury [24] and metabolic syndrome, which is associated with renal damage [25,26]. TAK-085 administration decreased TG levels in the kidney but not in the liver or plasma (Figure 2 and Table 1), suggesting that decreasing TG uptake from the plasma to the kidney or TG synthesis, or increasing TG degradation in the kidney may be involved. The same results were reported by Chin *et al.* [8]. They demonstrated that ω-3 PUFA prevents renal deterioration by attenuating SREBP-1 and reducing TG levels in the diabetic kidney. However, liver TG levels did not decrease following TAK-085 administration (Figure 2). These conflicting results could be because of cholesterol, which activates SREBP-1 via liver X-activated receptor α [27,28]. Because cholesterol levels in the liver were higher than those in the kidney, inhibition of SREBP-1 activity by TAK-085 may have been attenuated by cholesterol in the liver. In support of this finding, the stearoyl-CoA desaturase (SCD) index in the liver and kidney did not decrease following TAK-085 administration (Table 4 and Table S1) because SCD is transcriptionally upregulated by SREBP-1. EPA administration did not affect TG levels in the kidney (Figure 2). EPA also inhibits SREBP-1 induction [29,30]. Moreover, DHA affects SREBP-1c mRNA less markedly than EPA [31]. Thus, other factors such as lipase may be involved in decreasing TG levels in the kidney following TAK-085 administration. It has been reported that TG lipase mediated release of ARA for PG synthesis in rabbit kidney medulla microsomes [32]. In the present study, non-esterified ARA decreased in the kidney of the TAK-085-administered group (Figure 3), suggesting that TG lipase levels may decrease following TAK-085 administration; however, further experiments are necessary to clarify these points.

In the present study, RvEs, RvDs, and PD1 increased in the kidneys of the TAK-085-administered group and RvEs increased in the kidneys of the EPA-administered group (Figure 4 and Figure 5). RvEs, RvDs, and PD have recently been recognized as endogenous anti-inflammatory lipid mediators [33,34]. RvDs repress renal interstitial fibrosis, and PD1 promotes renoprotective HO-1 expression [35]. Administration of RvDs or PD1 to mice before ischemia results in a reduction in kidney injury [36]. Although inflammatory cytokines in the kidney were not measured in the present study, it has been reported that DHA and its lipid mediators RvD, PD1, and 14S,24R-dihydrooxy DHA decrease the levels of inflammatory cytokines in the kidney [17,37], suggesting that inhibition or resolution of inflammation in the kidney is involved in improving renal function and morphology. However, further experiments are necessary to clarify whether RvEs, RvDs, and PDs improve or prevent renal failure associated with metabolic syndrome. Although ω-3-derived lipid mediators bind specific receptors and provide anti-inflammatory actions [38], it is necessary to clarify whether these receptors are expressed in the kidney and whether their expression levels are affected by metabolic syndrome. Adiponectin upregulates hepatocyte chemokine-like receptor 1, also known as the RvE1 receptor, which is decreased in human fatty liver [39]. Thus, other mechanisms could be involved in improving renal function and reducing TG levels in the kidney.

Although ARA-derived eicosanoids play important roles in normal kidney functions, these eicosanoids are also involved in the pathogenesis of kidney disease [40]. PGI_2_ and PGE_2_ play critical roles maintaining blood pressure, renal function in a volume-contracted state, and renin secretion. Inhibiting COX may result in hypertension because of dysregulation of renal sodium excretion [41]. 5-Lipoxygenase (LO)-derived leukotrienes are involved in inflammatory glomerular injury. 12-HETE is associated with pathogenesis of hypertension, and may mediate angiotensin II and TGFβ induced mesangial cell abnormalities in diabetic nephropathy [42]. Here we demonstrated renal PGE_2_, 5-, 12-, and 15-HETE levels decreased in TAK-085-administred group (Figure 3) but blood pressure was not affected (Table 1). Taken together, these results suggest that TAK-085 contributes to adjust ARA-derived eicosanoid levels in the kidney and improves renal failure associated with metabolic syndrome.

It should be mentioned that our study had several limitations. The current results indicate that DHA-derived docosanoids are a central mediator to improve renal function in SHRcp rats, while, EPA-derived metabolites have anti-inflammatory effects as mentioned below, and we considered that these metabolites could have beneficial effects. Thus, in addition to the EPA group, TAK-085, combination of EPA with DHA, was selected in the current study; however, the present study did not have a DHA alone-administered group, which was the first limitation of our study. Further experiments are necessary to clarify whether administration of DHA alone improves renal function in SHRcp rats. The second limitation is that the direct effects of DHA-derived docosanoids on improvement in renal function in SHRcp rats were not assessed. By confirming this, it will become clear how important DHA-derived docosanoids are to improve renal failure associated with metabolic syndrome.

## 3. Experimental

All experiments were carried out in accordance with the Guiding Principles for the Care and Use of Animals in the Field of Physiological Science of the Physiological Society of Japan (2003) and approved by the institutional the Animal Care and Use Committee at Shimane University. 

### 3.1. Animals

Male SHR.Cg-*Lepr^cp^*/NDmcr (SHRcp, 6 weeks old) rats were used in this study. The animals were obtained from colonies of specific pathogen-free rats maintained by Japan SLC (Shizuoka, Japan). Housing conditions were thermostatically maintained at 23 ± 2 °C with constant humidity (50 ± 10%) and a 12 h-light/dark cycle (light on: 07:00−19:00). The animals were housed for at least 1 week before the experiments, and fed a fish-oil deficient diet containing 1% cholesterol and 0.3% cholic acid (Funabashi Farm Co., Chiba, Japan) and water *ad libitum*. The rats were divided into three groups by body weight, blood pressure, and plasma TG level: the TAK-085 group (n = 11), EPA group (n = 10), and control group (n = 10). TAK-085 and EPA group were orally administered TAK-085 (300 mg/kg body weight per day: Pronova BioPharma ASA, Oslo, Norway) containing 467 mg/g EPA, 365 mg/g DHA, and 3.8 mg/g α-tocopherol and EPA (300 mg/kg/day, Nisshin Pharma Inc., Tokyo, Japan) containing 980 mg/g EPA ethyl ester and 3.8 mg/g α-tocopherol emulsified in 5% gum arabic solution; the control group was orally administered 5% gum arabic solution containing 3.8 mg/g α-tocopherol once daily for 20 weeks.

### 3.2. Measurement of Blood Pressure

Arterial blood pressure and heart rate were determined by a tail cuff system at 2-week intervals. Briefly, conscious rats were lightly supported in a holder made of cloth mesh and maintained at 37 ± 1 °C (Model THC-1 Digital Thermo, Softron, Tokyo, Japan). Blood pressure from the tail artery was indirectly measured using tail-cuff apparatus (BP-98, Softron), which was controlled with a personal computer. Values are presented as the average of five independent measurements.

### 3.3. Blood Sample Assays

The blood was collected from retro-orbital plexus of rats every other week on the free feeding condition, and the plasma biochemical parameters were measured. Twenty-weeks after administration, rats were fasted for 12 h, collected blood, and measured the plasma biochemical parameters and fatty acids.

### 3.4. Analysis of Fatty Acid profiles

The fatty acid profiles of the plasma, and kidney, and liver homogenates were determined by gas chromatography, as described previously [43].

### 3.5. TG and Total Cholesterol (TC) in the Kidney and Liver

Liver and kidney was homogenized with phosphate buffer saline containing 0.05% BHT. Total lipids were extracted by Blay-Dyer method [44]. After dry down by N_2_ gas, then total lipids were reconstitute with 0.1% TritonX-100 in 2-propanol. TG and TC levels were determined by a kit (Wako Diagnostics, Osaka, Japan) according to the instructions.

### 3.6. ROS and Lipid Peroxidation (LPO) Measurement

ROS levels were measured as previously described previously [45]. Data are expressed as dichlorofluorescein production/min/mg protein. LPO levels were measured using the thiobarbituric acid reactive substance assay, as described previously [46], and data are expressed as moles of malondialdehyde/mg protein. Protein concentration was determined by the Lowry method [47].

### 3.7. Creatinine Clearance Measurement

Urine albumin and creatinine levels were measured using the Nephrat kit for the quantitation of rat urinary albumin and the Creatinine Companion kit (Exocell, Philadelphia, PA, USA) according to the manufacturer’s instructions. The ratio of the concentrations of albumin to creatinine (AC ratio) in urine was used as an index of urinary albumin excretion. Endogenous creatinine clearance (CrCl) was determined as CrCl = Ucr × V × Pcr^−1^, where Ucr and Pcr are urinary and plasma creatinine concentrations, respectively, and V is urine flow. The Ucr and V values were calculated from the data of SHR-cp rats in metabolic cages, and Pcr values were cited from Table 2. The CrCl was used as an index of glomerular filtration rate (GFR).

### 3.8. Morphological Analysis

Coronal sections of renal tissue (3–4 μm thick) were stained with periodic acid-Schiff (PAS) and examined by light microscopy in a blinded fashion. Glomerulosclerosis was evaluated semi-quantitatively according to criteria developed by Uehara *et al*. [48]. Briefly, 50 glomeruli were randomly selected from each animal for morphometric analysis. Glomerulosclerosis, defined as synechiae formation by PAS staining with focal or global obliteration of capillary loops, was graded as follows: 1+, <30% of glomerular area affected; 2+, 30% to 70% affected and 3+, >70% affected. The overall glomerular sclerosis score per animal was the average grade of all the glomeruli evaluated.

### 3.9. Analysis of Fatty Acid Metabolites

#### 3.9.1. Sample Preparation

Kidney homogenates were adjusted to 67% methanol and kept at −30°C, and samples were centrifuged at 5,000 ×*g* for 10 min at 4°C to remove precipitated proteins. The supernatants were diluted with ice-cold distilled water and adjusted to 10% (v/v) methanol. Internal standards (5 ng of PGE_2_-*d4*, PGD_2_-*d4*, PGF_2α_-*d4*, 5-HETE-*d8*, and ARA-*d8*) were added to each sample. Samples were acidified to pH 4.0 with 0.1 M HCl and were immediately applied to preconditioned solid-phase extraction cartridges (Sep-Pak C18, Waters, Milford, MA, USA) to extract the fatty acid metabolites. Sep-Pak cartridges were washed in succession with 20 mL water and 20 mL *n*-hexane. Finally, fatty acid metabolites were eluted with 10 mL methyl formate.

#### 3.9.2. LC-ESI-MS–MS-Based Analysis

Fatty acid metabolites in kidneys were measured, as described previously, with a slight modification [49,50,51]. High-performance liquid chromatography (HPLC) was combined with ESI–MS using a TSQ quantum mass spectrometer (Thermo Fisher Scientific K.K., Tokyo, Japan). HPLC was performed using a Luna 3u C18(2) 100Å LC column (100 × 2.0 mm, Phenomenex, Torrance, CA, USA) at 30 °C. Samples were eluted in a mobile phase comprising acetonitrile–methanol (4:1, v/v) and water–acetic acid (100:0.1, v/v) in a 27:73 ratio for 5 min, ramped up to a 100:0 ratio after 25 min, and held for 10 min at a flow rate of 0.1 mL/min. MS–MS analyses were conducted in negative ion mode, and fatty acid metabolites were detected and quantified by selected reaction monitoring (SRM). Conditions for the detection of each compound by SRM are listed in Supplemental Table 2. Peaks were selected and their areas were calculated using the Xcalibur 2.1 software (Thermo Fisher Scientific K.K.).

### 3.10. Statistical Analysis

Results are expressed as means ± standard errors. Data were analyzed with a one-way ANOVA with Dunnett’s post hoc test. Differences between the groups were considered significant at *P* < 0.05. All statistical analyses were performed using PASW Statistics 18.0 (IBM-SPSS, Inc., Armonk, NY, USA).

## 4. Conclusions

The results of our study show that the use of combined therapy with DHA and EPA in SHRcp rats, an animal model of renal dysfunction with metabolic syndrome, improved or prevented renal failure associate with metabolic syndrome by decreasing TG levels and increasing the levels of the lipid mediators RvEs, RvDs, and PD1. 

## Supplementary Materials

Supplementary materials can be accessed at: http://www.mdpi.com/1420-3049/19/3/3247/s1.
