# Supplementary material for: Omega-3 Fatty Acids Protect Renal Functions by Increasing Docosahexaenoic Acid-Derived Metabolite Levels in SHR.Cg-Leprcp/NDmcr Rats, a Metabolic Syndrome Model
Source: Molecules. 2014 Mar 17;19(3):3247–63. doi: 10.3390/molecules19033247 (PMC6271130; doi:10.3390/molecules19033247)
Supplement: Supplementary file 1 [file molecules-19-03247-s001.pdf]

Supplementary Material

Table S1. Effects of TAK-085 and EPA on fatty acid profiles in the liver.

(mol%)	Control	TAK-085	EPA
PLA (16:0)	29.97 ± 0.18	28.28 ± 0.17 *	28.44 ± 0.27 *
STA (18:0)	3.88 ± 0.20	3.75 ± 0.13	4.03 ± 0.25
OA (18:1n-9)	43.31 ± 0.52	40.77 ± 0.43 *	40.15 ± 0.70 *
LA (18:2n-6)	16.12 ± 0.21	17.37 ± 0.29 *	17.05 ± 0.25 *
ALA (18:3n-3)	0.66 ± 0.02	0.96 ± 0.03 *	0.87 ± 0.02 *
ARA (20:4n-6)	4.49 ± 0.30	2.66 ± 0.09 *	3.25 ± 0.26 *
EPA (20:5n-3)	0.23 ± 0.01	1.45 ± 0.05 *	2.58 ± 0.12 *
DPA (22:5n-3)	0.47 ± 0.03	1.16 ± 0.06 *	2.31 ± 0.12 *
DHA (22:6n-3)	0.73 ± 0.08	3.47 ± 0.13 *	1.17 ± 0.07 *
n-6/n-3	9.98 ± 0.34	2.87 ± 0.08 *	2.96 ± 0.08 *
DHA/ARA	0.16 ± 0.01	1.32 ± 0.07 *	0.37 ± 0.01 *
EPA/ARA	0.06 ± 0.01	0.55 ± 0.03 *	0.83 ± 0.06 *
SCD index	11.53 ± 0.66	11.02 ± 0.41	10.43 ± 0.76

Note: PLA, palmitic acid; STA, stearic acid, OA, oleic acid; LA, linolenic acid; ALA, α-Linolenic acid; ARA, arachidonic acid; EPA, eicosapentaenoic acid; DPA, docosapentaenoic acid; DHA, docosahexaenoic acid. SCD index was estimated as ratio of OA to STA. Values are means ± SE for 10–11 rats. *, Statistically significant from control group ($P < 0.05$, Dunnett t-test).

Table S2. Selected reaction monitoring (SRM) transitions of fatty acid metabolites.

Compound	SRM Transition (*m/z*)	Compound	SRM Transition (*m/z*)	Compound	SRM Transition (*m/z*)
ARA	303 > 259	EPA	301 > 257	DHA	327 > 283
PGE_2	351 > 271	5-HEPE	317 > 115	7-HDoHE	343 > 141
PGD_2	351 > 271	12-HEPE	317 > 179	10-HDoHE	343 > 153
$PGF_{2\alpha}$	353 > 193	15-HEPE	317 > 219	13-HDoHE	343 > 193
5-HETE	319 > 115	18-HEPE	317 > 259	14-HDoHE	343 > 205
12-HETE	319 > 179	RvE2	333 > 115	17-HDoHE	343 > 245
15-HETE	319 > 219	RvE3	333 > 213	PD1	359 > 153
ARA-d_8	311 > 267			RvD1	375 > 141
PGE_2-d_4	355 > 275			RvD2	375 > 175
PGD_2-d_4	355 > 275				
$PGF_{2\alpha}$-d_4	357 > 197				
5-HETE-d_8	327 > 116				

Note: ARA, arachidonic acid; PG, prostaglandin; HETE, hydroxyeicosatetraenoic acid; EPA, eicosapentaenoic acid; HEPE, hydroxyeicosapentaenoic acids; Rv, Resolvin; DHA, docosahexaenoic acid; HDoHE, hydroxydocosahexaenoic acid; PD1, Protectin D1.
